# Interdisciplinary perspectives on privacy awareness in lifelogging technology development

**DOI:** 10.1007/s12652-022-04486-5

**Published:** 2022-12-12

**Authors:** Wiktoria Wilkowska, Julia Offermann, Liane Colonna, Francisco Florez-Revuelta, Pau Climent-Pérez, Alex Mihailidis, Angelica Poli, Susanna Spinsante, Martina Ziefle

**Affiliations:** 1 Human-Computer Interaction Center, RWTH Aachen University, Aachen, Germany; 2 Swedish Law and Informatics Research Institute, Stockholm University, Stockholm, Sweden; 3 Department of Computer Technology, University of Alicante, Alicante, Spain; 4 Department of Occupational Science and Occupational Therapy, University of Toronto, Toronto, Canada; 5 Department of Information Engineering, Marche Polytechnic University, Ancona, Italy

**Keywords:** Lifelogging applications, Privacy, Acceptance, Interdisciplinary project

## Abstract

Population aging resulting from demographic changes requires some challenging decisions and necessary steps to be taken by different stakeholders to manage current and future demand for assistance and support. The consequences of population aging can be mitigated to some extent by assisting technologies that can support the autonomous living of older individuals and persons in need of care in their private environments as long as possible. A variety of technical solutions are already available on the market, but privacy protection is a serious, often neglected, issue when using such (assisting) technology. Thus, privacy needs to be thoroughly taken under consideration in this context. In a three-year project PAAL (‘Privacy-Aware and Acceptable Lifelogging Services for Older and Frail People’), researchers from different disciplines, such as law, rehabilitation, human-computer interaction, and computer science, investigated the phenomenon of privacy when using assistive lifelogging technologies. In concrete terms, the concept of *Privacy by Design* was realized using two exemplary lifelogging applications in private and professional environments. A user-centered empirical approach was applied to the lifelogging technologies, investigating the perceptions and attitudes of (older) users with different health-related and biographical profiles. The knowledge gained through the interdisciplinary collaboration can improve the implementation and optimization of assistive applications. In this paper, partners of the PAAL project present insights gained from their cross-national, interdisciplinary work regarding privacy-aware and acceptable lifelogging technologies.

##  Introduction

Today, structures and processes which have been stable for decades in many countries require rethinking on different levels. Societies are faced not only with political and economic upheavals connected to concerns around climate change and digitization, but also with structural and health-related changes like the ones currently caused by the COVID-19 pandemic. Such disruptions are shaping our lives now and will shape our future. In addition, many countries are experiencing a significant increase in the number of older citizens (65 + years) in the wake of demographic change. To maintain a healthy equivalence between the younger and the older part of the population, it is necessary to appropriately counteract the consequences arising from the demographic imbalance by addressing chronic illnesses and disabilities in a cost-efficient manner and respecting the needs of frail and sick persons as well as their caregivers (Mihailidis and Colonna 2020). To mitigate the social and economic effects of aging, current technology developments in the medical sector offer far-reaching opportunities. Assisting technologies in terms of Ambient Assisted Living (AAL) as well as diverse lifelogging applications allow for meaningful support in a wide variety of areas (for an overview see Rashidi and Mihailidis 2013; Blackman et al. 2016). In private settings, older persons and individuals with long-term illnesses or impairments can be supported in their everyday life by increasing their medical safety, e.g., via the detection of emergencies and falls (e.g., Mubashir et al. 2013) and the monitoring of vital parameters (e.g., Rashidi and Cook 2009) as well as daily habits and activities (Poli et al. 2020a). The latter also enables identifying changes in behavior, movement patterns, sleep pattern, or walking speed, eventually allowing the recognition of indicators for diseases such as dementia or Parkinson’s disease (e.g., Hayes et al. 2008; Suzuki et al. 2007). Emergency and fall detection in turn can be realized in different ways, i.e., as wearable technologies (Lai et al. 2010), as sensor-based or microphone-based technologies (Zigel et al. 2009), radar and depth-based technologies (Cippitelli et al. 2017) or even as video-based technologies and systems (Climent-Pérez et al. 2020).

According to these approaches, support and assistance can be provided in both private settings and professional care contexts. The use of assisting technologies in care environments can serve to address current challenges, such as the lack of care personnel and the increasing numbers of people in need of care, by providing relief and support in tasks of the daily care routine (Rashidi and Mihailidis 2013). Here, the preventive application of nighttime wandering represents an example for relieving formal caregivers by detecting and alerting in case of deviation from predefined routes or normal behaviors (e.g., Kim et al. 2009; Fudickar and Schnor 2009).

Although the approaches are largely discussed for many application fields, all these developments have in common that they are predominantly based on one specific (technical) discipline, having a limited view and restricted perception of the overall topic. Indeed, in order to implement a broad spectrum of assisting lifelogging systems—fulfilling diverse supporting and relieving functions, as well as being both privacy-aware from a legal perspective and sustainably accepted by their future users—an interdisciplinary collaboration of diverse technical, legal, and social disciplines is needed.

For this reason, the current work describes a multidisciplinary view on using lifelogging technologies for assistance in the everyday life which was the primary goal of the European project PAAL (‘*P*rivacy-*A*ware and *A*cceptable *L*ifelogging services for older and frail people’). A team of lawyers, psychologists, engineers, computer and communication scientists from Sweden, Spain, Italy, Germany, and Canada, integrating crossing disciplines to share different types of knowledge and different perspectives, have developed assisting lifelogging services specifically tailored to the needs and requirements of older users. In addition to the increased awareness of ethical, legal, social, and privacy issues associated with utilizing lifelogging technologies, PAAL researchers aimed at evaluating technology acceptability issues and barriers to familiarity with technology in order to develop possible strategies for overcoming them. The motivation in this paper is thus to present multidisciplinary research perspectives—as opposed to the former, rather unilaterally oriented research—that bundles up the multiple expertise and during the project gained knowledge regarding the topic of privacy awareness in lifelogging technology.

## Privacy by design as a legal requirement

Privacy is a nebulous concept subject to a countless number of understandings. Attempts to define privacy in a legally coherent way have generated an immense body of scholarship (Burdon 2010). Some commentators, referred to as privacy reductionists, do not even think that there should be a distinct legal right to privacy since the right derives from other rights such as liberty, contract or property interests (Thomson 1984; Nissenbaum 2010). The reductionists contend that legal claims to privacy should be resolved by other areas of law and by failing to do so these other rights are degraded (Nissenbaum 2010). Generally, however, most scholars agree that the concept of privacy is an integrated, distinct, and coherent right (Gavison 1980; Nissenbaum 2010). The problem becomes that they have a wide range of views on what is precisely distinctive about the values that fall under the rubric of privacy (Nissenbaum 2010).

Privacy is generally considered essential to human wellbeing, development, creativity, mental health, liberty, dignity, emotional release, self-evaluation, and inter-personal relationship of love, friends, and trust (Solove 2008; Nissenbaum 2010). It is also considered to be a necessary condition for autonomy insofar as it provides the space for individuals to experiment in life and develop their own personality and thoughts, without being subject to the constant judgment of others (Nissenbaum 2010). In addition to furthering individual values, privacy also brings many benefits to society as a whole to the extent that it nourishes and promotes the values of a liberal, democratic, political, and social order (Regan 1995).

As of today, despite the immense amount of thoughtful scholarship on the subject, there is no single coherent theory regarding the right to privacy. Solove has set forward six categories of privacy concepts in his well-known article *Conceptualizing Privacy*: (1) the right to be let alone, (2) limited access to the self, (3) secrecy, (4) control over personal information, (5) personhood, (6) intimacy (Solove 2002; Gormley 1992). More recently, Nissenbaum has set forward a theory of privacy as “contextual integrity” where she contends that the right to privacy is neither a right to secrecy nor a right to control but a right to “appropriate flow” of personal information (Nissenbaum 2010).

From the perspective of European human rights law, the privacy concept was first incorporated into the legislative framework in the 1950s, when the Council of Europe signed the Convention on Human Rights (ECHR, all the member states of the EU are also signatories of the European Convention on Human Rights). Article 8 of the ECHR provides a right to respect for one’s “private and family life, his home and his correspondence,” subject to certain conditions (Council of Europe, CoE, 1950). The fundamental rights set forth in the Convention, including the right to privacy, were gradually acknowledged in the jurisprudence of the Court of Justice of the European Union (CJEU) as constituting general principles of EU law, stemming from the common constitutional traditions of the Member States.

In 1981, the Council of Europe adopted the Convention 108 which secures the right to privacy as enshrined in Article 8 of the ECHR in regard to automatic processing of data by safeguarding the individual against the unjustified collection, processing, use, storage, and dissemination of their personal data. The Convention 108 became the foundation for the EU Data Protection Directive which was adopted in 1995 to regulate the collection, processing and transfer of personal data within the EU (European Parliament 1995). In 2000 the EU proclaimed its own instrument of fundamental rights protection, the Charter of Fundamental Rights of the European Union, without however giving it legally binding effect. Article 7 of the Charter reiterates the definition of privacy given by the ECHR (European Union 2012a). Additionally, Article 8 of the Charter specifies that “everyone has the right to the protection of personal data concerning him or her” (European Union 2012b). With the entry into force of the Lisbon Treaty on 1 December 2009 the Charter of Fundamental Rights became legally binding and was recognized as having the same legal value as the Treaties.

On 14 April 2016, the EU adopted the General Data Protection Regulation (GDPR) which aims to “harmonize data privacy laws across Europe, to protect and empower all EU citizens data privacy and reshape the way organizations across the region approach data privacy.” The GDPR repeals the Data Protection Directive, representing a stronger and more coherent data protection framework for the Union. In 2018, the CoE modernized its Convention 108 to reflect advances in data protection rights, particularly brought forward by the GDPR.

Within this broader theoretical and substantive legal context, the concept of Privacy by Design (PbD) has emerged as an approach to assure that privacy concerns are addressed at the outset of a technology’s development. It evolved from the concept of Privacy Enhancing Technologies (PETs), which refers to a variety of technology-driven solutions that seek to strengthen the protection of personal data in information and communication technologies (ICT) by preventing the unlawful collection, use, and disclosure of personal data (Yee 2011). While PETs are solely focused on technology, PbD also includes organizational measures designed to respond to legal requirements. Organizational measures include matters, like conducting privacy impact assessments, documenting data processes that contain personal data, and appointing a Data Protection Officer (DPO). PbD is a recognition that technology alone is insufficient to ensure adequate protection of privacy: The requirements of privacy laws should be embedded into both organizations and systems. Cavoukian, Information Commissioner of Ontario, largely credited for establishing the concept, explains PbD as a “systematic approach to designing any technology that embeds privacy into the underlying specifications or architecture” (IPC 2009).

When the GDPR became enforceable beginning 25 May 2018, PbD shifted from a theoretical, policy goal (nice-to-have) to a binding legal requirement called Data Protection by Design (DPbD), placing a data controller at risk for substantial fines for noncompliance (Jasmontaite et al. 2018). DPbD is governed by Article 25 of the GDPR which explicitly requires data controllers to “implement appropriate technical and organizational measures… which are designed to implement data protection principles…, in an effective manner and to integrate the necessary safeguards into [data] processing” (GDPR 2016). Recital 78 provides a list of potential measures that may assist a data controller with its compliance burden, such as minimizing the processing of personal data, pseudonymizing personal data as soon as possible, providing transparency with regard to the functions and processing of personal data, enabling the data subject to monitor the data processing, and enabling the controller to create and improve security features (Waldmann 2019). In addition to organizational measures and PETs, Article 25 mandates the use of Data Protection by Default (PbDf), meaning “that in the default setting the user is already protected against privacy risks” (ENISA 2014).

Implementing technological and organizational safeguards to guarantee the protection of personal data is a critical legal requirement, especially because these devices process highly sensitive personal data like key human biological signals that, if lost or stolen, will not only put an individual’s reputation at risk, but can also threaten his or her health or wellbeing. However, because of the nascent nature of the industry and the not-so-specific commercial identification of *lifelogging products* or *services*, there is a lack of guidance for systematically embodying values like privacy into lifelogging systems (e.g., Wiese Schartum 2016; Mulligan and King 2012; Spiekermann 2012; Alshammari and Simpson 2012). That said, in the project PAAL, a methodology for reliably embodying values like privacy into lifelogging systems has been developed, pushing the bounds of available approaches. It starts with conducting an analysis for the contextual understanding of privacy, especially the privacy threats and risks posed by the particular lifelogging technology (Mihailidis and Colonna 2020); an overview of key privacy concerns raised by lifelogging technologies is provided in the Appendix. Here, theoretical and empirical studies conducted by experts in human-computer relations are necessary in order to understand how privacy functions in the lives of people affected by the systems in question (Flanagan et al. 2008). Next, it is necessary to evaluate the relevant normative framework, specifically identifying legal rules, and setting forward a systematic approach as to how the requisite black-letter law can be incorporated into lifelogging devices.

Third, it is key to consider the specific design elements of lifelogging systems which include at least the following levels: the sensors, the models, the system, the user interface, and the user. The sensor level considers the raw data that forms the basis for further analysis. The model level examines theories about the dataset. At the system level, personal data are processed according to the model. The user interface level considers how data is displayed and the user level considers how a user can take actions based on the data (Mihailidis and Colonna 2020). Fourth, the identification of DPbD techniques, strategies, and patterns that can serve as potential responses to legal requirements must take place. These techniques will involve various degrees of sophistication, efficacy, and expense. DPbD techniques should be implemented in order to meet legal requirements.

## Realization of PbD approach using exemplary lifelogging applications

To address privacy awareness of lifelogging technologies, we elaborate on two exemplary applications which are meant to support individuals with frail health condition to manage their everyday lives and maintain their autonomy. To fulfill data protection rules, we approach these applications on the basis of five different levels as defined above, considering the technical design and the technology users. The first application addresses a preventive lifelogging application, enabling prompting and reminding functions for frail or disabled users (e.g., dementia patients). The prompting and reminding system (PRS) integrates different technologies such as a speech-based system that reminds the users of their daily activities (e.g., food and fluid intake, hand washing), as well as an installed depth camera to monitor the presence of the user in a specific area. Collecting the user’s information from this lifelogging system enables providing prompts and reminders and identifying early changes in health or behavior. The second application focuses on the recognition of activities of daily living (ADL), which can be performed by using wearable cameras, cameras located in the environment, and sensors embedded in mobile phones or smart wristbands. The addressed daily activities refer to basic self-care tasks (e.g., bathing and showering, personal hygiene, dressing, functional mobility, self-feeding) and instrumental activities (e.g., cleaning and maintaining the house, managing money, preparing meals, shopping, taking medications, communication and moving within the community). Each application context was separately evaluated in two independent empirical studies.

### Prompting and reminding systems

We use a voice assistant (VA) as a PRS example. VA is an emergent technology and has gained widespread attention across the world (Al-Heeti 2019; Bohn 2019; Gartner 2016; Malkin et al. 2019). It allows for lively interactions and makes people feel like chatting with a real person (Luger and Sellen 2016; Nass et al. 1999).

#### Sensor level

VA can be used for different purposes, such as monitoring a frail person’s food intake and reminding a person to take medication, and going to a doctor’s appointment (Bian et al. 2021; Cofre et al. 2020). It records a person’s voice and people around him/her having a conversation, which could reveal the identity of the person and the people that he/she is interacting with (Nautsch et al. 2019). Therefore, the recordings impose significant privacy concerns (Gurrin et al. 2014). Furthermore, these recordings could be reviewed and analyzed by a human being rather than a computer program for device quality control (Liao et al. 2020). As such, this speech data has the potential to be exposed to snoopers and hackers (Nautsch et al. 2019; Prabhakar et al. 2003). Thus, VA could be intrusive to personal privacy (Prabhakar et al. 2003). Here, methods that potentially maximize the privacy protection from the user’s perspective are needed (see Sect.  3.1.4). Considering the risk of the potential exposure of personal privacy that can lead to breach or misuse of sensitive data, an alternative is to use less privacy intrusive sensors that provide reminders and monitor changes in a person’s health status. For example, fridge-door sensors could be used to monitor nutrition intake of a person with dementia (PWD) by observing the number of times the fridge was used and prompt the user to take food when necessary (Bian et al. 2021). Motion sensors and wearable sensors could be used to measure physical activity and prompt the user to do exercises (Bian et al. 2021; Mukhopadhyay 2014).

#### Model level

With the popularity and wide use of mobile devices, it is an obvious option to utilize them to assist individuals and make their life easier and more comfortable (Silver 2019; Silver et al. 2019). As mentioned above, reminder applications have been developed to help PWD maintain autonomy as long as possible. Studies on effectiveness of the mobile health (mHealth) apps have also reported that these apps could potentially increase physical activity (PA) among older adults (Aslam et al. 2020; Muellmann et al. 2018).

#### System level

At a system level, multiple sensors can be combined to provide richer and reliable information of a person to achieve the PRS goal. A standalone sensor/technology, due to limited information it can provide, may not provide accurate information and all other necessarily required information. To ease the concern of privacy intrusion, a less privacy intrusive sensor network could be applied to monitor a person’s life providing prompts and reminders to the user and/or his/her caregivers as needed. Using a contact sensor as an example, the device is not able to differentiate people living in the same household. Only using the contact sensor to report, for instance, the number of times a person uses the fridge to determine their nutrition intake would be misleading (Bian et al. 2021). In addition, the person’s uses of the fridge may not necessarily mean they are getting food (i.e., get water or drinks from the fridge) (Bian et al. 2021). An improved method could be combining a Radio Frequency Identification (RFID) tag, a camera, a smartphone, and a contact sensor to obtain more valuable information. The RFID is to identify the person of interest who uses the fridge. The camera is used to monitor what food is taken out from the fridge. If the user’s food intake deviates from the usual intake, a prompt would be sent to the smartphone of the user to remind them to eat food. In this case, the use of the camera would also be more acceptable as it does not reveal a person’s identity but only the food from the fridge (Bian et al. 2021). Only passively monitoring a person’s PA using technology, such as a wearable or motion sensor, may not effectively motivate a person for exercise automatically. Studies show that sending reminders (i.e., text message reminder or reminder app) to the mobile device could encourage and motivate a person to do PA (Kim and Glanz 2013; Müller et al. 2016). Therefore, combining the wearable/motion sensor and the mobile device (i.e., smartphone) could produce better results for exercise. As smartphones are gaining popularity (Silver 2019; Silver et al. 2019), it makes sense to integrate them with the PRS. However, using smartphones also poses some privacy risks (Temming 2018): For example, hackers could hack into the phone and track a person’s location. They could hack a medication reminder app and send a “take medication” command. Such actions could cause privacy breaches and result in negative consequences for the person’s life. Hence, it is vital to take action proactively to avoid privacy breaches (see Sect. 3.1.4).

#### User interface level

We propose some methods that could potentially maximize the privacy protection of the PRS, focusing on the users‘ interaction with the applications. The company that is commercializing the product should provide a valid, high standard privacy policy that can be easily accessed by the users (Liao et al. 2020). A privacy policy is a document that entails the data practice of an organization or developer (Liao et al. 2020). A good and useful privacy policy should inform users what data is being collected, how their data is stored, used, and shared, and who has access to the data (Liao et al. 2020). The privacy policy should be concise, and easy to be understood by the end users (Bonilla and Martin-Hammond 2020; Liao et al. 2020). The company should adopt the Privacy and Security by Design approach, take actions proactively to protect user’s privacy, and respect for individual’s privacy (Cavoukian 2009b; Cavoukian and Dixon 2013). Also, the company should have a strict policy for third app developers to publish apps on their platform, such as thoroughly review the app privacy policy and only allow app developers that provide a valid and good quality privacy policy to use the platform (Liao et al. 2020).

From the developer’s side, they should embed a valid standard privacy policy into their apps (Liao et al. 2020; Sunyaev et al. 2015). A study researching the privacy policy of mHealth apps found that the app privacy policy was often not available (Sunyaev et al. 2015). Among apps with privacy policy, the privacy policy was poorly written, was not written in lay language, and the content was often not specifically app-related, which could result in loss of interest in reading it for users (Liao et al. 2020; Sunyaev et al. 2015).

Developers should keep privacy as a priority when designing technology and privacy should be embedded into the technology design (Cavoukian 2009b). For example, applying certain privacy-preserving techniques within the device, such as homomorphic encryption (HE) and secure two-party computation (STPC) (Nautsch et al. 2019). It is a best practice that developers apply Privacy by Design at all levels of processing components when designing technology (Cavoukian 2009b; Nautsch et al. 2019). The end users could also maximize their privacy, using following practices (Federal Trade Commission 2020; Quain 2019):

- Before using the technology, carefully review the company’s privacy policy and understand how the data is being used, where and how long the data will be stored, and who may have the authority to access the data;
- Turning off or mute the device when it is not used;
- Set up the device to automatically delete the past recordings or manually delete the recordings periodically;
- Create a strong password and apply multi-factor authentication when setting up the account;
- Secure Wi-Fi network, such as applying encryption on a network.

Further, choose a non-identifiable technology for PRS to remind a person’s ADL, such as reminder apps. In a case study with a mild PWD of El Haj et al. (2017), the authors found that compared to the baseline phase the user completed more target tasks with the Google calendar reminder during the intervention phase. In another study, McGoldrick and her colleagues (2019) investigated the MindMate app, a reminder tool to help prompt the events for mild PWD. The results showed that the app is effective in reminding PWD’s daily activities.

Thus, both organizations and developers should put user’s privacy as a priority. But also users should proactively take action to protect their privacy and the risks connected with its breach. The Office of the Privacy Commissioner of Canada (2016) recommends for the users following measures for a mobile device:

- Keep the device in a secure place and prevent it from stealing;
- Set up a strong password and encrypt the device whenever is possible;
- Secure home Wi-Fi network by using a strong password and hide the network name. Remove the auto-connect function on the mobile device so that the device has always manually connected to a network;
- Use a Virtual Private Network (VPN) in a public area;
- Carefully review the privacy policy of the apps or programs before installing. Make sure to understand what data may be collected and if any sensitive information will be obtained and stored, where and how long data will be stored, and who may have the authority to access the data;
- Install apps or programs from trusted websites. Keep programs updated and remove outdated apps.

#### User level

At the user level, a user-centered study empirically evaluated user acceptance of the prompting and reminding system (PRS). A sample of *N* = 176 participants remained for statistical analyses after data cleansing procedures. The participants were on average 34.6 years old (*SD* = 13.7; *min* = 17; *max* = 88; *median* = 29) and 60.8% (*n* = 107) were female (male 39.2%, *n* = 69). With regard to the health status, only 16.5% (*n* = 29) reported to suffer from chronic diseases. Thus, the entire sample consisted of rather young and healthy individuals.

**Fig. 1:**
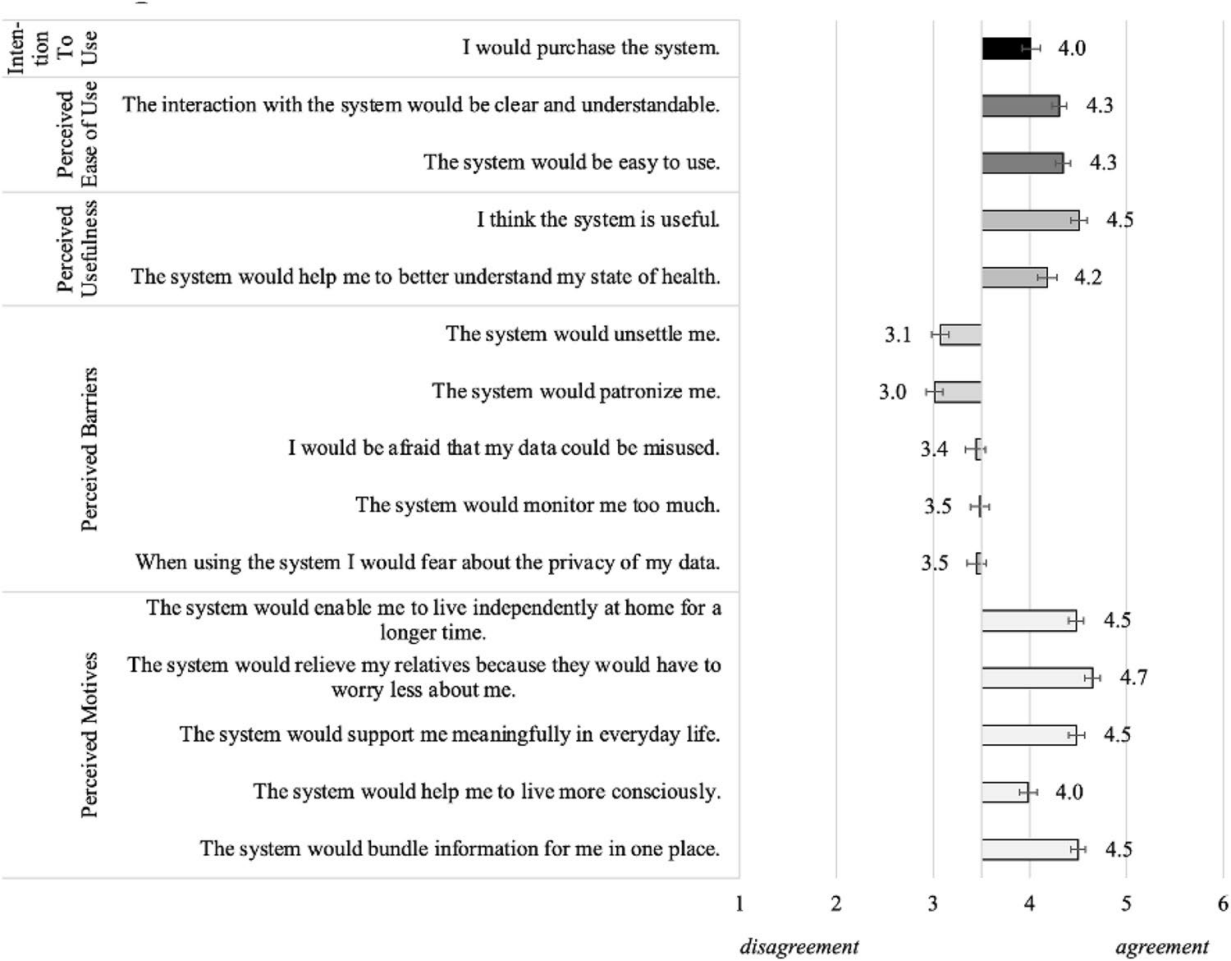
Acceptance and perception (means and standard errors) of PRS lifelogging application (*N* = 176)

*Method.* In order to reach a broad sample of (future) lifelogging technology users, an online survey was conducted aiming for an investigation of the acceptance and perception of PRS lifelogging technologies. The participants assessed the key acceptance constructs of the Technology Acceptance Model (TAM, Davis 1989): Intention to use, Perceived Usefulness (2 items, Cronbach’s α = 0.76), and Perceived Ease of Use (2 items, α = 0.84). In addition, perceived motives (5 items, α = 0.86) and barriers (5 items, α = 0.87) of using the PRS lifelogging application were also evaluated in order to consider aspects such as privacy concerns, as they have proven to be relevant for future users in previous qualitative interview studies. All assessed items of the described constructs can be seen in Fig. 1 and were evaluated each on six-point Likert scales.

**Fig. 2:**
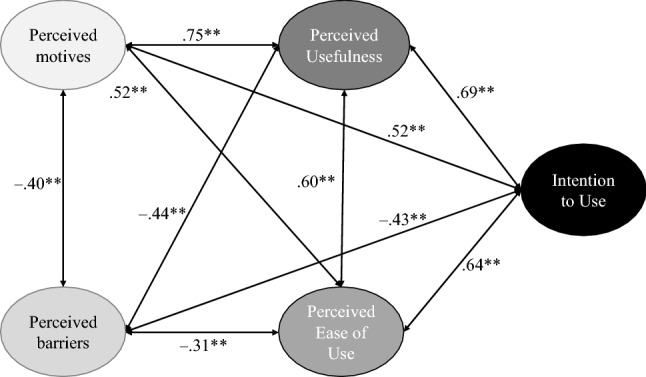
Relationships between acceptance and perception constructs (correlation coefficient *r*, **p < 0.01)

*Results.* Overall, the participants’ evaluations showed a positive Intention to Use the PRS lifelogging application (*M* = 4.0; *SD* = 1.3). In addition, also all items regarding the Perceived Usefulness (e.g., “I think the system is useful”: *M* = 4.5; *SD* = 1.1) and Perceived Ease of Use (e.g., “The system would be easy to use”: *M* = 4.3; *SD* = 1.0) received approving ratings by the participants. Items referring to the perceived barriers of using the system were evaluated neutrally up to slightly rejective, while fears with regard to ‘privacy’ (*M* = 3.5; *SD* = 1.3), ‘too much monitoring’ (*M* = 3.4; *SD* = 1.4) and a potential ‘misuse of data’ (*M* = 3.4; *SD* = 1.4) were most relevant. Considering the perceived motives to use the system, all items received approving ratings, whereas the benefit of ‘relieving relatives’ (*M* = 4.7; *SD* = 1.1) was most, and the support ‘to live more consciously’ (*M* = 4.0; *SD* = 1.2) least, important for the participants. An additional correlation analysis (see Fig. 2) showed that the Intention to Use the PRS application was strongly connected with Perceived Usefulness and Perceived Ease of Use, but also with the perceived motives, and moderately related with the perceived barriers of using the PRS. Further, the Perceived Usefulness was strongly positively correlated to the perceived motives and moderately negatively connected with perceived barriers of using the PRS. A regression analysis revealed that up to 52.4% (adjusted *r*^*2*^) of the Intention to Use the PRS can be predicted by the four constructs referring to the technology acceptance, i.e., Perceived Usefulness, Perceived Ease of Use, as well as the Perceived Motives and Barriers of using the PRS.

*Summary of the study.* The results of this exemplary study show the importance of considering other (besides from conventional models known) acceptance factors, such as motives and barriers expressed and considered to be important by the future users, as they have a high potential to impact the users’ acceptance and adoption of an innovative technology or system. Within the project PAAL, we therefore realized an interdisciplinary and iterative exchange between the legal, technical, and social perspectives on lifelogging technology development in order to address, consider, and integrate the users’ requirements and wishes adequately.

### Recognition of activities of daily living (ADL)

In the domain of technological approaches aiming at the automatic recognition of ADL, it is common to distinguish between solutions that rely on the use of cameras (video-based ones) and/or depth sensors (in addition to microphones that are usually integrated in cameras; Pires et al. 2018; 2019), and sensor-based solutions without visual information (e.g., Gambi et al. 2020; Wu et al. 2019). In this section, we consider two approaches: The former describes visual information that is exploited for automatic recognition of ADL and proper privacy-preserving solutions (3.2.1). The latter considers non-visual approaches, focusing on the use of wearable and ambient sensors to point out how privacy-related issues may still be present, despite the apparently low degree of personal information generated by the aforementioned types of sensors (3.2.2). Eventually, an empirical study evaluates the user acceptance of such technology for ADL recognition (3.2.3).

#### Approaches based on visual information

**Fig. 3:**
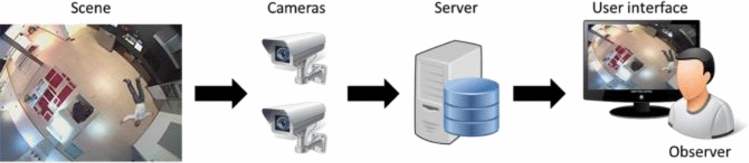
Typical architecture of a video surveillance system

As stated by Senior (2009), there are several locations in which visual privacy preservation can be performed in an architecture of a vision-based monitoring system (Fig. 3). In this architecture, data would flow from the video cameras to the user interfaces, passing through the video processor and the database. In newer systems, the processing can be done in the cloud, which carries a greater risk for the preservation of privacy. To solve this issue, many systems consider local processing, e.g., having the processor/server in the home and transferring only concealed data to the cloud or to the observer. In this regard, Padilla-López et al. (2015) and Ravi et al. (2021) review different mechanisms to protect privacy in video data.

*Sensor level.* There are different approaches to protect visual privacy at the sensor level, i.e., avoiding that private information is acquired or broadcast by the video-based device:

1. Intervention methods deal with the problem of preventing someone to capture private visual data from the environment (Padilla-López et al. 2015). These methods physically interfere with camera devices to prevent the acquisition of an image by means of a specialized device that interferes with the camera optical lens. For instance, a Bluetooth transmitter could be worn by a person who wants their privacy protected. The camera might stop recording when this transmitter is in its proximity, ensuring that private images are not taken. This can also be done by providing the user the possibility of turning off the cameras at their discretion. These methods would, in most cases, be usable when using environmental cameras.
2. Embedding in the camera the algorithms to recognize ADL. Therefore, no image will be broadcast. Richardson (2012) proposed a Descriptive Camera that works like a normal camera, in the sense that users aim at what they want to capture. But, instead of producing an image, in some minutes it outputs a text description of the scene provided by a Mechanical Turk worker. Of course, this is not an automatic process and, therefore, privacy is not preserved as the worker has access to the images. Recent advances in the video-based recognition of ADL (Climent-Pérez et al. 2020) as well as image and video captioning (Krishna et al. 2017; Hossain et al. 2019) might lead soon to obtain relevant descriptions automatically.
3. Use of depth or thermal data only: Most of the systems that require the acquisition of image data in private environments substitute cameras or RGB-D sensors by depth or thermal sensors, acquiring only information about the distance or the temperature of the different objects in front of the device. For instance, Pramerdorfer et al. (2020) presented a commercial depth sensor for monitoring residents in elderly care facilities and alerting caretakers in case of dangerous situations such as falls or residents not returning to their beds at night.
4. Reduction of the image resolution: Tao et al. (2019) combined the use of low resolution and thermal data to protect the users’ privacy. An 8 × 8 infrared sensor array can detect the occurrence of falls and activities of daily living, while retaining user visual privacy. Ryoo et al. (2017) followed a similar approach by using 16 × 12 and 32 × 24 RGB images of the environment. This dimensionality reduction might also be obtained by employing an auto-encoder to output a representation in the latent space.

*Model level.* An alternative to environmental cameras is to mount a camera on the user’s head or torso and record activities from an egocentric perspective, i.e., from the subject’s own point of view (Nguyen et al. 2016). As stated by Fathi et al. (2011), there are three main reasons why the egocentric paradigm is particularly beneficial for analyzing activities that involve object manipulation. First, occlusions of manipulated objects tend to be minimized, as the workspace containing the objects is usually visible to the camera. Second, since poses and displacements of manipulated objects are consistent in workspace coordinates, objects tend to be presented at consistent viewing directions with respect to the egocentric camera. Third, actions and objects tend to appear in the center of the image and are usually in focus, resulting in high quality image measurements. However, the continuous video recording of every experiential moment—whether at home, at work, around family, or in public spaces—involves not only those doing the recording, but anyone who happens to be recorded. Egocentric vision has greatly enhanced the vulnerability of bystanders (Ferdous et al. 2017). Recent research has worked on preserving visual privacy of the third parties that did not give consent: Dimiccoli et al. (2018) analyzed how image degradation might preserve the privacy of persons appearing in the image while activities can still be recognized; Hassan and Sazonov (2020) proposed an image redaction approach for privacy protection by selective content removal using a semantic segmentation-based deep learning.

*System level.* When it comes to the system level privacy of end products and services, no unique implementation exists and many times it comes down to the companies’ own policy, vision, and business model (e.g., user profiling in exchange for cheaper products or services). For instance, some commercial solutions offer a security section on the websites, where they state how they enforce privacy and security policies regarding the user data. However, when reading their legally binding texts, they require overseas transfer of data outside EU territory, processing and storing the video data gathered from their products in the US. In recent years, whistle-blowers have proven how “systematic” these law enforcement requirements can be, providing access to camera footage to a foreign (third party) government, rendering GDPR protections useless. Some manufacturers directly explain that they transmit, process, and store the video streams provided by the end users of their products and services, and email in plaintext snippets of those streams (i.e., sensitive information sent over a non-encrypted protocol), to notify the end user when relevant events happen (e.g., motion detected, intruders, etc.). The Guidelines 3/2019 on processing of personal data through video devices set out the limitations of video data processing for companies and have a specific household exemption (European Data Protection Board 2019). However, this is not a blanket protection for any camera in the house, since these must be aimed at usage “in the course of a purely personal or household activity”, “and is not clearly the case with the processing […] publication on the internet […] accessible to an indefinite number of people”. Also, it cannot cover “even partially, a public space”. Furthermore, “the user of video surveillance at home needs to look at whether he has some kind of personal relationship with the data subject, whether the scale or frequency of the surveillance suggests some kind of professional activity on his side, and of the surveillance’s potential adverse impact on the data subjects”. This means that cameras installed in the house by assisted living companies would not fall under the exemption.

**Fig. 4:**
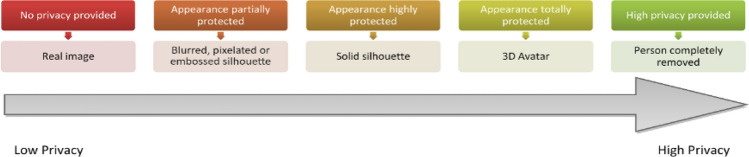
Different levels of privacy according to the observer and their relationship to the observer; The left-most level offers the view of the full unprocessed image, aimed for the users themselves or very close relatives; As levels lay more to the right, visual privacy is increased, e.g., changing the person for a 3D avatar, which still retains semantics of the scene, but better preserves identity

*User interface level.* Chaaraoui et al. (2014) introduced a privacy-by-context approach, in which elements that constitute the identity of a user are recognized. With these, it is then possible—using different visualizations as shown in Fig. 4—to adapt the privacy level on the basis of the relationship of the user and the observer, as well as considering other cues which make up the context: (a) *Identity* of the user to retrieve their preferences; (b) *Appearance* (e.g., clothing, partial, or full nudity); (c) *Location* (e.g., kitchen, bathroom, bedroom, etc.); (d) *Ongoing activity* (e.g., cooking, watching TV, etc.); (e) *Event*: what happened during this (i.e., fall, loss of consciousness, alarm button pressed); (f) *Observer*: to determine whether they have access rights; (g) *Relationship* (i.e., relative, health processional, caregiver, friend, etc.); and (h) *Response* by the subject (if requested).

**Fig. 5:**
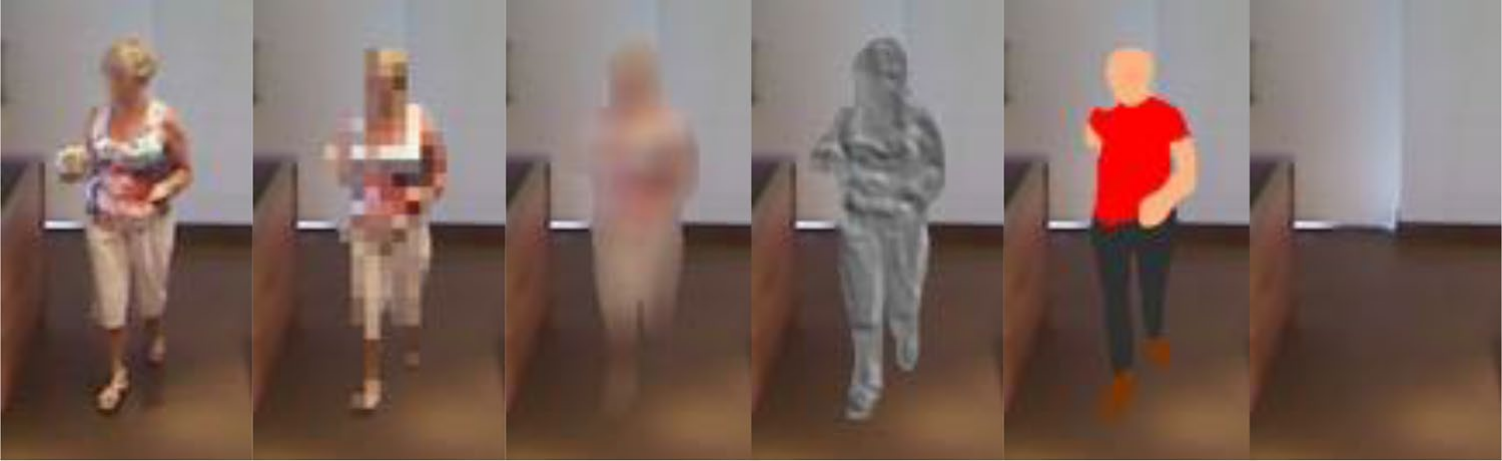
Example frame from the Toyota Smarthome dataset (Das et al. 2019) and the application of different visualization filters; From left to right: original image, pixelation, blurring, embossing, replacement with an avatar, person removal

Using such a context-aware scheme with different levels of data protection, it is possible to obtain tailored visualizations for different stakeholders. Privacy can be preserved, while maintaining the necessary intelligibility required for each application and observer. There must be a trade-off between those two components of a privacy filter (i.e., intelligibility vs. usefulness of the data). A recent work by Climent-Pérez and Florez-Revuelta (2021) implements this privacy-by-filter approach to images acquired with RBG cameras in home environments (Fig. 5).

#### Approaches based on non-visual information

**Fig. 6:**
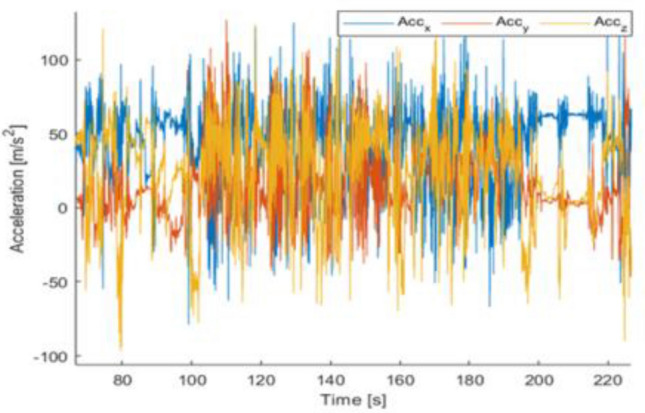
Time evolution of the acceleration along the three spatial directions, measured by a wrist-worn device applied to a lady performing the „washing dishes“ ADL

*Sensor level.* Thinking of wearable devices and non-audiovisual ambient sensors to collect data aimed at automatic ADL recognition, it may appear that the level of intrusion into the privacy of the monitored subject could be so limited to become negligible. In fact, recognition of ADL by means of wearable devices typically involves collecting acceleration signals (Hussain et al. 2019; Hegde et al. 2018) that do not explicitly expose personal or identifying characteristics of the person to whom the sensor is attached. As an example, Fig. 6 shows the time evolution of the acceleration along the three spatial directions x, y, and z, collected from a wrist-worn device used by an old lady during the execution of the “washing dishes” ADL. A visual inspection of the signal does not provide any specific clue about the gender, age or other individual characteristics of the person performing the activity. Nevertheless, the acceleration signals contain enough features to allow the automatic classification of the performed ADL (Poli et al. 2020b; Sridharan et al. 2020).

**Fig. 7:**
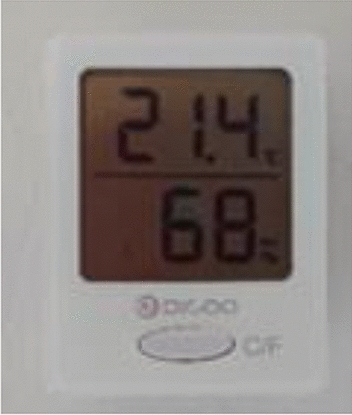
A temperature and humidity sensor for home monitoring

A similar condition happens when looking at ambient sensors that collect binary information, such as on/off, in/out, presence/no presence, or sensors that provide a measure of a given quantity, such as temperature, humidity, and light sensors. If not provided with its corresponding semantic description, each of the aforementioned sensors does not incur in privacy leakage. A quite simple but explanatory example may be given with a temperature and humidity sensor, like the one shown in Fig. 7, that does not apparently provide any other information than the values of the two measured quantities which can be read from its display.

**Fig. 8:**
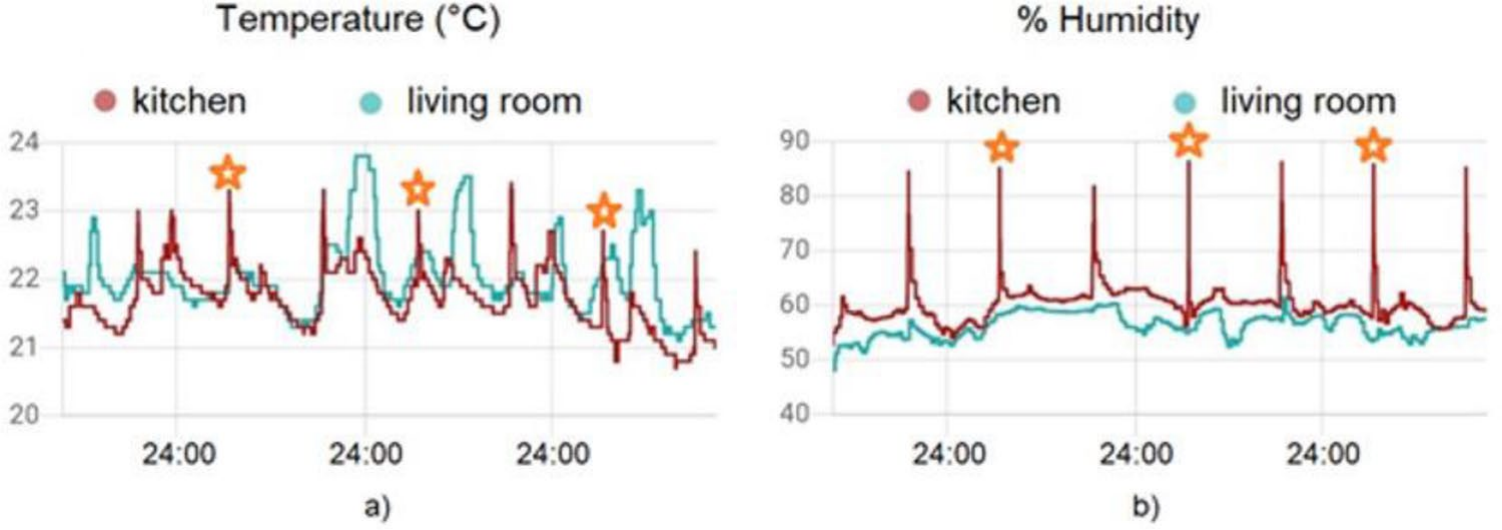
Times series over three days of the **a** temperature and **b** humidity measured by the sensor (shown in Fig. 6) inside two different rooms of the same house; Orange stars, periodically located around noon each day, identify temperature and humidity peaks reasonably associated to “cooking lunch” ADL

The situation changes if we add contextual details to the values measured by the sensor: For example, knowledge about its location in the home (in the kitchen, in the bedroom, or in a different room) provides a useful starting point to extract meaningful and potentially exploitable information from the sensor data. By collecting the sensor measurements over a given time frame (a day, a week, or more) and adding the contextual label, it is possible to scan the sequence of measurements and look for repetitive patterns that may allow to infer sensitive information, related, for example, to the living habits of the person in the home. Continuing with the example above, adding the contextual label „kitchen“ to the data measured by the temperature and humidity sensor shown in Fig. 7, and looking for repetitive patterns in the temperature and humidity time series, it becomes much easier to assume that the person living in the home usually has lunch at around noon and probably cooks something (maybe pasta?), given the sharp increase in the humidity values. This is visible in the graphs shown in Fig. 8.

In essence, it is a combination of basic sensor information collected from the home premise, timestamp, and contextual information that must be setup „a priori“ allowing for the automatic recognitions of ADL by means of very simple yet widespread sensors inside of living environments, as experimentally shown by Matsui et al. (2020).

As long as acceleration data from wearable devices and measurement data from ambient sensors can be accessed without their associated contextual labels, the risk of exposure of private details may be considered low. Nevertheless, the access to data time series may determine privacy losses under temporal correlations, as interestingly discussed and analyzed by Niu et al. (2019). In order to protect sensor data from unauthorized access and exploitation, encryption-based solutions could be envisioned, but in most of the cases they result incompatible with the power and computational capabilities available onboard ambient sensors, unless such security mechanisms have been accounted for since the original design. Dynamic key management for symmetric encryption would be another quite difficult task to solve as well (Perez-Jiménez et al. 2019). A quite recent approach, not yet so much widespread in the market, is based on the Integrated Circuit Metric (ICMetric) technology that exploits features of a device to generate an identification, which is then used for the provision of cryptographic services (Tahir et al. 2018). An alternative way to address the problem of protecting the user’s privacy, which is being investigated in the framework of the PAAL project, focuses on identifying those features inherently present within the collected signals, like in the case of the acceleration measured from a wrist-worn device, with the aim of removing those features, which may expose personal details about the person, without hindering the performance of automatic ADL classification algorithms (Poli et al. 2021).

*Model level.* Acceleration measurements from sensors attached to the body or carried in pockets have been proved able to expose identification details about the person. In fact, Gafurov et al. (2007) have shown how gait recognition may be performed from the signals measured by a body-worn sensor and exploited for authentication purposes. Gait has been defined as a behavioral biometric (Abuhamad et al. 2021) as it shows distinct patterns for every individual, and higher order statistics computed from gait are exploited in subject identification (Sprager and Juric 2015). Within the PAAL project, researchers investigated whether by subjects performing different types of ADL acceleration signals collected from the wrist may release personal details similarly to gait signals, which could be exploited to identify the person‘s gender or age. In such a case, proper de-identification algorithms should be applied to acceleration signals to limit the unintentional release of personal details while enabling, at the same time, the automatic ADL recognition. A Random Forest (RF) classifier, fed with time- and frequency-domain features computed over acceleration signals measured on the wrist from subjects performing six different types of ADL, was trained on signals obtained from young subjects and tested on signals collected from an older adult. Findings showed that features computed over the single directional components of the acceleration are significant for the aim of age discrimination and more informative than those computed over the acceleration magnitude, confirming that acceleration signals from the wrist are quite different in young and elderly subjects due to some physical limitations in old age. This could reveal personal information about the observed subject’s age range, even if the same is not actually relevant for the aim of specific applications (Poli et al. 2020c).

*System level.* Addressing PbD at the system level of wearable and ambient sensors requires a complex and deep integration among several different components: the device, the wireless communication interface, the data collector, and the remote server receiving the transmitted data, which is typically a cloud-based one. As an example, it is useful to check the privacy policy available for the Empatica E4 wearable device used by the PAAL project to carry out lifelogging-related research (Empatica 2021). The actors/roles involved in the data collection process are identified as: the *device* and its *applications* (including but not limited to the Research Portal, the E4 Manager, the E4 Real Time, and the E4 Server) connected to the device as purchased by the *user*, in order to provide the *services* offered by Empatica. Empatica is the data controller of the personal data collected from the user through the device and the app, and it does not have access to the final users wearing the device.

*User interface level.* The nature of wearable devices leaves little space for interaction between the device and users. Mohzary et al. (2020) proposed to add a privacy-aware layer over a sample smartwatch Operating System (O.S.) to limit user data access through the enforcement of user-set privacy settings and a specific interface is designed to capture the user’s preferences. Kim et al. (2020) analyzed privacy concerns raised by collection of an individual’s health data through wearable devices, and proposed a method based on local differential privacy to aggregate the collected health data in a privacy-preserving manner. Within the PAAL research, the Empatica E4 device (Empatica 2021) is used as a wrist-worn sensor that allows to access measured signals in their raw format.

#### User level regarding applications for recognition of activities of daily living

A further empirical study within the PAAL project focused on the perceptions of personal and data privacy when using lifelogging technologies for ADL. Data from *N* = 209 participants aged between 18 and 79 years (*M* = 37; *SD* = 15.1) were finally considered for statistical analyses. The sample was well-balanced in gender (54% female, *n* = 112; 46% male, *n* = 97) and most participants (81%; *n* = 170) reported to be in good health condition.

*Method.* Using a standardized online survey, we examined participants’ general attitude towards using, and their intention to use, technologies that are able to lifelog their daily basic and instrumental activities (e.g., sensors in mobile phones or wristbands, environmental and portable cameras). We also asked the respondents to assess their perceptions of personal privacy and data security when utilizing such applications. Scales used in the survey partially validated items from some forerun studies (Schomakers and Ziefle 2019; Wilkowska and Ziefle 2012) and partially resulted from preliminary interviews, which explored privacy awareness as regards the use of lifelogging technologies. The scales evaluated the following constructs: (i) *general attitude* towards using lifelogging for ADL (3 items; Cronbach’s α = 0.91), (ii) *intention to use lifelogging* for different ADL (e.g., body hygiene, mobility, nutrition, medication intake, etc.), and (iii) perceptions of personal *privacy* (5 items; Cronbach’s α = 0.71) and data *security* (3 items; Cronbach’s α = 0.71). For assessments of the particular constructs, we used four- and six-point Likert scales as presented in the figures below.

**Fig. 9:**
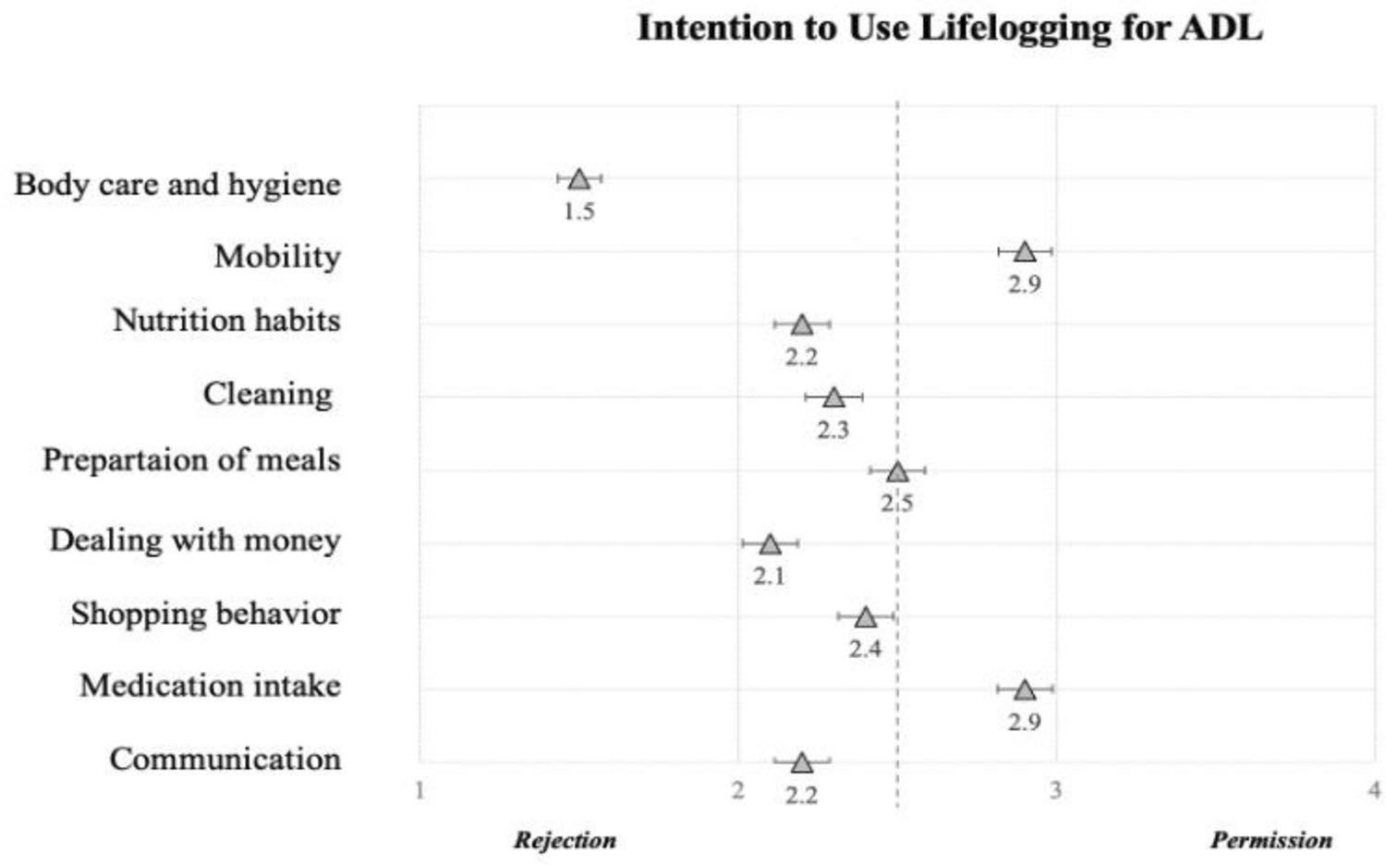
Intention to use lifelogging technologies for ADL (*N* = 209); based on Wilkowska et al. (2021b)

**Fig. 10:**
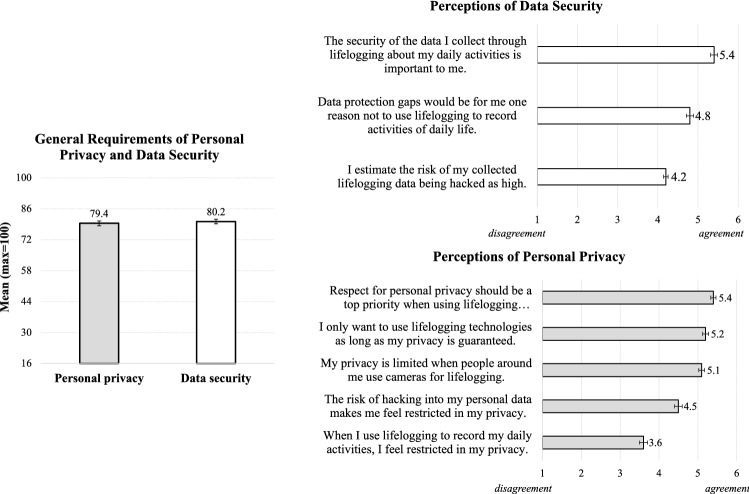
Perceptions of personal privacy and data security when using lifelogging technologies for activities of daily living (*N* = 209): overall scales (left) and individual items of the constructs (right)

*Results.* Considering the general attitude towards using lifelogging for ADL (e.g., “I consider it beneficial to record my daily activities using lifelogging technology.”), our study revealed a slightly positive attitude among the participants who reached a mean of *M* = 11.2 (*SD* = 3.5; *min* = 3, *max* = 18) points on the agreement scale. Even more detailed results emerged regarding the intention to use lifelogging technologies for ADL: Here, the majority of the resulting means oscillated around the middle of the scale between permission and rejection. Participants approved lifelogging for their mobility behavior (*M* = 2.9, *SD* = 1.2) and medication intake (*M* = 2.9, *SD* = 1.2), and they neutrally assessed to lifelog their preparation of meals (*M* = 2.5, *SD* = 1.2), while other applications were rated as rather undesirable (Fig. 9). Moreover, statistical analyses revealed that participants had high expectations for their personal privacy (*M* = 79.4; *SD* = 14) and data security (*M* = 80.2; *SD* = 15.4). As depicted in Fig. 10, the overall scales (left) reached high means and most responses on the individual item level (right) reached high agreement on the importance of personal privacy and data security when using lifelogging for ADL.

*Summary of the study.* Findings of this study empirically corroborate the relevance of privacy awareness in utilizing lifelogging technologies assisting (frail) persons in their daily activities in private environments. At the user level, personal privacy and data protection are perceived as top priorities of the use. However, respondents significantly differentiate their assessments depending on the particular technology (e.g., sensors vs. cameras) and the life-logged activity. Here, future work is necessary to make more precise specifications.

## Discussion

In this paper, we exemplarily present two types of lifelogging tools used for PRS and the recognition of ADL, which were evaluated within the interdisciplinary project PAAL. We focus on minimizing the risk of privacy breach caused by using these devices. We also suggest alternative methods employing “quantified self”-sensors, such as wearable devices, which are less likely to pose major risks to privacy (Gurrin et al. 2014). In the following, we firstly discuss the key insights and elaborate guidelines for the development of privacy-aware lifelogging solutions. Subsequently, we present the progress and lessons learned from the project collaboration.

### Project insights and guidelines for development of lifelogging solutions

There are different types of technology for PRS and ADL. Technologies such as VA and mobile devices (i.e., smartphones) could cause considerable privacy concerns. Despite the risk of significant privacy invasion, VA and smartphones still attract our interest as users and have gradually become integrated as part of our lives. The adoption rate of these devices is growing rapidly (Al-Heeti 2019; Bohn 2019; Gartner 2016; Malkin et al. 2019; Silver 2019; Silver et al. 2019), and it is inevitable that more and more of these technologies will be used either independently or in combination with other technologies to support autonomous living and aging-in-place. Because of the rapid increase in their popularity, these devices are still immature and in their early investigational stage regarding privacy implications. Therefore, it is urgent to involve all stakeholders (i.e., organizations, developers, government, and users) and put every effort into proactively protecting user privacy. Organizations should proactively protect user data (Cavoukian 2009b; Cavoukian and Dixon 2013). A first step would be to develop a valid and high standard privacy policy that is readily available and accessible for users (Liao et al. 2020). In reality, however, privacy considerations have not been addressed by organizations and developers as they should be (Liao et al. 2020; Sunyaev et al. 2015). In general, organizations tend not to review the content of privacy policies with sufficient care (Liao et al. 2020; Sunyaev et al. 2015). It was often found that the URL link to the privacy policy was broken, the content of the policy was irrelevant to the specific app, a different privacy policy was used for the same app published on different platforms, and the privacy policy was not revised with each update (Liao et al. 2020; Sunyaev et al. 2015). Additionally, the content of the privacy policy is not transparent to the users (Liao et al. 2020; Malkin et al. 2019) or lacks a clear statement on whether the data would be reviewed and accessed by other people (Liao et al. 2020; Malkin et al. 2019).

Notwithstanding this, such apps are still allowed to be published on the platforms. Therefore, privacy protection should start at the level of business practices (Cavoukian and Dixon 2013). The PbD approach should be set as a continuous goal for an organization and should be followed consistently through the business practices (Cavoukian and Dixon 2013). Studies from the end users’ perspective have demonstrated that users are concerned about their privacy when using digital technology (Bian et al. 2020; Bonilla and Martin-Hammond 2020; Malkin et al. 2019). For example, users worry about the VA being able to record their conversations that entail sensitive and/or embarrassing information (Malkin et al. 2019). Although studies show that users do not have a clear understanding of how VA works, and how their data would be processed (Chandrasekaran et al. 2018; Lau et al. 2018; Malkin et al. 2019; Zheng et al. 2018), this technology is still rapidly growing in popularity. It was found that users lack awareness that they can control their privacy, i.e., delete their recordings or turn off the VA (Malkin et al. 2019). In addition, users are not accustomed to reading the privacy policy before installing an app. They would still download the apps even without a valid policy link provided (Liao et al. 2020; Sunyaev et al. 2015). However, this behavior could be changed by public education from the company, community, and/or social media to raise potential users’ awareness to privacy control and privacy risks (Cavoukian 2009a; Office of the Privacy Commissioner of Canada 2018). To build up trust and make people feel confident in using digital technology, governments need to act proactively in order to protect the user’s privacy. Also, governments should work closely with organizations to assist and guide them in designing products with a properly embedded privacy protection. In summary, a robust privacy protection framework requires the effort and involvement of government, organizations, developers, and users (Office of the Privacy Commissioner of Canada 2018; 2019).

### Résumé of the project progress

The project PAAL provides the initial framework for considerations on using lifelogging technologies for daily assistance of older and frail persons, supporting their private and institutional caregivers. In the time frame of the project, experts from different disciplines were able to effectively elaborate and intertwine the important outcomes. As a basis for accepted and to the users’ needs optimally adjusted technical solutions, in the first step ethical and legal aspects included in the concept of PbD were combined with the exploration and validation of social requirements connected to the use of assisting technologies (Mihailidis and Colonna 2020). Based on this framework, technical conceptualizations iteratively took place considering concurrently conducted empirical approaches that modelled technology acceptance—especially for the older and frail technology users—and sounded out for the perceived issues to the familiarity with the applied lifelogging technologies.

Using a multi-leveled approach enables a holistic and comparative way of technology development and offers effective ways to better support the older segment of society, counteracting the associated economic consequences within the healthcare system. The collaborative project PAAL demonstrated such an approach, (1) providing legal regulations and socio-ethical recommendations in order to accordingly protect the user’s privacy from abuse, (2) appropriately considering the real user’s needs, perceived benefits, barriers, and conditions of an accepted use as revealed in empirical studies, and (3) consequently implementing the gained knowledge into the technical realization.

### Lessons learned from the interdisciplinary collaboration

The interdisciplinary exchange during the PAAL project enabled a multi-facetted holistic development of assistive lifelogging technologies. From the legal perspective, Privacy by Design has arisen as a core mechanism to address the complex privacy challenges that result from the use of such technologies. At the same time, research revealed a lack of clarity surrounding how precisely to embed privacy into systems. In an effort to approach this research gap, the interdisciplinary collaboration has provided a number of examples as to how privacy can be methodologically integrated into various levels of lifelogging systems, particularly taking technological, legal, and social perspectives into account.

From the user perspective, the active involvement of the (potential) users in all phases of the project has proven to be a fundamental part of the holistic and interdisciplinary process. Findings revealed positive attitudes towards diverse lifelogging technologies, acknowledging especially the health-related assistive functions. The user acceptance resulted to be more reluctant for the video-based in comparison to the sensor-based applications. However, during the project we also identified significant cultural differences, which should be also considered in the product design and development (Wilkowska et al. 2021a; Offermann-van Heek et al. 2020). The gained insights should be addressed not only in projects, but especially in commercial uptake of lifelogging technologies for frail and older users.

From the technical perspective, our approach enabled a holistic, complementary, and comparative way of technology development. While the multi-leveled technical approach for identifying the PbD issues in lifelogging may be generalized to different technologies, ranging from VAs to video-based systems and from wearable to ambient sensor-based technologies, to implement the actual privacy countermeasure is strongly solution-specific.

Concerning wearable devices and non-audiovisual ambient sensors, the exposure of private details can be considered low at the sensor level, especially if measurement data can be accessed without the associated time and contextual information. However, the manufacturers of devices must declare the privacy policy according to the GDPR rules (i.e., at the system level) to inform the user. Additionally, a de-identification algorithm can be applied at the model level to ensure and increase whatsoever data protection, while enabling the automatic recognition of ADL. As a further lifelogging application, the development of vision-based intelligent systems has enabled not only streaming video in real time, but also extracting useful information from visual data to analyze actions, activities, and behaviors. At this point, it is worth noting that in AAL applications (as we pointed out in Sect. 3.2.1) user identification might not be an issue, as the identity of the user is already known and, in most cases, only one person would be present (e.g., an older person living alone). Concerns are more related to the disclosure of appearance (e.g., if the person is dressed/naked) and behavior. With the recent advances in deep learning for classification and recognition, it is also needed to protect privacy, not only from people who could get access to the images, but from machine learning algorithms that could extract private information from the images. Ravi et al. (2021) classify machine obfuscation methods into poisoning attacks and evasion attacks. Poisoning attacks aim to disrupt the training of machine learning models by introducing specific “poisoned” images so that the models behave in unexpected ways. Evasion attacks transform the acquired images in a way that they are difficult for image recognition systems to be identified.

However, even with the latest breakthrough with deep learning techniques, the video-based systems for lifelogging applications are not completely reliable. Therefore, these systems must currently be semi-supervised, which means that the final assessment should be performed by a caregiver, once a detection or a log is performed by the automatic system. Here, a compromise between privacy preservation and intelligibility of the data is required in order to conceal private data and, at the same time, be able to assess the situation. If the video does not need to be broadcast, it can be replaced by other imagery data, such as depth, thermal, or low-resolution data. How these types of data will aggravate the assessment of inexperienced (non-tech savvy) caregivers, should be focused on in future research.

## Conclusion and outlook

In the light of some serious privacy concerns that threaten the potential of lifelogging applications to improve efficiency and care in the healthcare settings, there is a high demand for a robust privacy protection framework which requires the active involvement of governments, organizations, developers, and users. The collaboration in the presented project PAAL provides a framework combining legal, social, user-centered, and technical requirements for the design of lifelogging technologies, being used for the daily assistance of older and frail people as well as their private and institutional caregivers. Besides, the presented empirical findings and the interdisciplinary exchange reassert that privacy concerns can pose a serious deterrent to the technology adoption. An interdisciplinary and multi-technological approach in the development enables thus more promising, competitive, and sustainable technology solutions for the users in the future.

## Appendix

**Declarations**.

## Data Availability

The datasets generated and analyzed for the purposes of the project are not publicly available due to the sensitive data of participants but are available from the corresponding author on reasonable request and with permission of the funding organizations.

## Appendix

See Table 1.

**Table 1:** Key privacy concerns raised by lifelogging technologies

Key attributes of lifelogging technologies that present special challenges to privacy (Mihailidis and Colonna 2020)	
Embedded nature of lifelogging devices	- Lifelogging devices are integrated into the environment making it challenging for the user to, among other things, fully understand the surveillance capabilities of the device - These devices are often embedded in the home which is an area that is traditionally given a very high level of privacy protection, especially parts of the home like the bathroom or bedroom - Many other lifelogging devices can be worn directly on the body or even implanted into the flesh which intrudes into an even more sacred area of privacy—the human body (Colonna 2021)
Context awareness	- Lifelogging devices are able to recognize an individual user and situations. A lifelogging device may assist an older adult who is living alone in her home by giving this individual context-aware reminders regarding unattended activities (e.g., it may remind the individual to take her medicine or to refill the toilet paper in the bathroom (Cavoukian et al. 2010)) - Through collecting raw data, modelling these data, and reasoning about context, lifelogging devices can understand an enormous amount about an individual’s private life - Not only can these devices understand matters like where an individual is located and whom an individual is with but they can also understand what resources are nearby
Personalized nature of lifelogging	- Gives rise to privacy concerns, as well as broader concerns about profiling and discrimination - While lifelogging devices can provide tailored services to an individual, this often requires the use of artificial intelligence and machine learning techniques fueled by large quantities of personal data which create complex privacy concerns - Personalized services often cannot be created without at least relying on a pseudo-identity to which a user profile can be attached, limiting the ability to utilize privacy preserving anonymization techniques - Other features of lifelogging devices that give raise to privacy concerns include their opacity, adaptive nature, their ability to anticipate behavior, and their autonomous nature
**Privacy concerns raised by lifelogging devices** (**Colonna 2019a, Colonna 2019b)**	
The ubiquity and invisibility of lifelogging devices	- Privacy concern about whether consent can be used as a ground to lawfully process personal data under the GDPR. It is challenging to obtain a data subject’s unambiguous consent in advance of the data processing because the potential uses of the data and insight gained from the processing are difficult to predict and explain ex ante - It is very challenging to obtain fully informed consent because most devices lack screens and therefore, it is hard to display a privacy policy - Obtaining consent from individuals who are frail or sick and perhaps suffer from diminished cognitive abilities is a huge challenge, not least because the mental capacity to consent appears on a spectrum (Batchelor et al. 2012)
The non-consensual logging of third parties or bystanders	- Lifelogging devices are seamlessly entrenched in living environments, making it very easy to capture image, speech, or location data about third parties without these individuals having any awareness that the sensors even exist
Data accuracy (particularly the accuracy of the underlying data collected by the sensors and the accuracy of the algorithms and techniques applied in the data processing)	- A core issue is that lifelogging technologies “collect their data from the real world using imperfect sensing devices.” (Branch 2013) - Sensors themselves might have faults or errors due to things like incorrect sensor installation (Quevedo et al. 2017) - External factors like power instability and temperature changes could further endanger the quality of the data collected by a lifelogging device - When it comes to the algorithms that process the data running through a lifelogging device, it is well-established that if the training data are biased, incorrect, incomplete or labelled incorrectly then the model will reflect and perpetuate the bias. (Cofone 2019)
Security issues	- Security vulnerabilities can cause harm to the private life of an individual as well as threaten their physical health and well-being - Medical records and sensitive health data are prime targets of hackers since they have high black market value and can be misused to obtain medical care - Furthermore, these devices typically lack a user interface, which makes authentication through, for example, a username/password problematic - Lifelogging device can continue to collect data, even after the battery of the device dies (Troiano 2017), which can then be “stored within vulnerable network systems, the security of which is largely, if not entirely, unregulated.” (Arnow 2016) - Finally, lifelogging devices can be integrated which increases the risk of data breach since “the least secure device becomes the security level for all [the devices].” (Kellogg 2016)
The issue of data sensitivity	- Not only does lifelogging in the health context involve the processing of highly sensitive data like health data, but it also involves the creation of some extremely sensitive data points such as a person may be suffering from cognitive impairment
Complex data flows (Colonna 2019a)	- Lifelogging technologies involve data flows whereby key biological signals are collected as well as incidental and behavioral data such as location, patterns, and related metadata. These data, high in quantity and sensitivity, can be combined and re-combined to discover “new” data that an individual may have no consciousness about whatsoever - The individual may be completely unaware of this data and thereby unable to contest its accuracy, control its flow or to challenge its use by others
“Black-box medicine”	- Lifelogging devices and the digital ecosystems they feed into can be very opaque in the ways they handle data - Nicholson Price refers to “black-box medicine” as “the use of opaque computational models to make decisions related to health care.” (Price II 2017) - Some “black boxes” are entirely opaque to humans, while others can be reverse engineered or probed to determine a loose ranking of the importance of the variables the AI takes into account.” (Bathaee 2018)
Pernicious surveillance and pernicious memory (Allen 2008)	- Pernicious surveillance concerns the routine and systematic collection about an individual’s existence by a lifelogging device which can be used for purposes like marketing, security and, social control - Pernicious memory refers to the use of lifelogging devices to collect a 24/7, never-ending record of an individual’s life, even where it clear there are some moments of an individual life that are best forgotten
Additional information (References)	Allen AL (2008) Dredging Up the Past: Lifelogging, Memory, and Surveillance. University of Chicago Law Review 75: 47–74. Arnow G (2016) Apple Watching You: Why Wearable Technology Should Be Federally Regulated, Loyola of Los Angeles Law Review 49: 607–634 Batchelor R, Bobrowicz A, Mackenzie R, Milne A (2012) Challenges of Ethical and Legal Responsibilities When Technologies’ Uses and Users Change: Social Networking Sites, Decision Making Capacity and Dementia. Ethics and Information Technology 14: 99–108 Bathaee Y (2018) The Artificial Intelligence Black Box and the Failure of Intent and Causation, Harvard Journal of Law and Technology 31: 889–938 Branch J W, Giannella C, Szymanski B, Wolff R, Kargupta H (2013) In-network Outlier Detection in Wireless Sensor Networks, Knowledge and Information Systems 34: 23–54 Cavoukian A, Mihailidis A, Boger J (2010). Sensors and in-home collection of health data: A privacy by design approach. Information and Privacy Commissioner of Ontario, Canada. Cofone IN (2019) Algorithmic Discrimination Is an Information Problem. Hasting Law Journal 70: 1389–1444. Colonna L (2019a) In Search of Data Protection’s Holy Grail Applying Privacy by Design to Lifelogging Technologies. In: Leenes R, Hallinan D, Gutwirth S, De Hert P (eds.) Data Protection and Privacy: Data Protection and Democracy. Hart Publishing, pp. 173–207. Colonna L (2019b) Legal and Regulatory Challenges to Utilizing Lifelogging Technologies for the Frail and Sick, International Journal of Law and Information Technology 1: 50–74. Colonna L (2021) Artificial Intelligence in the Internet of Health Things: Is the Solution to AI Privacy More AI? Boston University Journal of Science and Technology Law 27: 312–343. Kellogg S (2016) Every Breath You Take: Data Privacy and Your Wearable Fitness Device, Journal of The Missouri Bar 72: 76–82. Price II WN (2017) Regulating Black-Box Medicine, Michigan Law Review 116: 421–474. Quevedo J, Garcia D, Puig V, Saludes J, Cugueró MA, Espin S, … Valero F (2017) Sensor data validation and reconstruction. In: Real-Time Monitoring and Operational Control of Drinking-Water Systems (pp. 175–193). Springer, Cham. Troiano A (2017) Note, Wearables and Personal Health Data: Putting a Premium on Your Privacy, Brooklyn Law Review 82: 1715–1753.
